# Relationships between human vitality and mitochondrial respiratory parameters, reactive oxygen species production and dNTP levels in peripheral blood mononuclear cells

**DOI:** 10.18632/aging.100618

**Published:** 2013-11-30

**Authors:** Scott Maynard, Guido Keijzers, Martin Gram, Claus Desler, Laila Bendix, Esben Budtz-Jørgensen, Drude Molbo, Deborah L. Croteau, Merete Osler, Tinna Stevnsner, Lene Juel Rasmussen, Flemming Dela, Kirsten Avlund, Vilhelm A. Bohr

**Affiliations:** ^1^ Center for Healthy Aging at the University of Copenhagen, 2200 Copenhagen N, Denmark; ^2^ Department of Cellular and Molecular Medicine at the University of Copenhagen, 2200 Copenhagen N, Denmark; ^3^ Danish Aging Research Center, Universities of Aarhus, Southern Denmark and Copenhagen, Denmark; ^4^ Department of Biomedical Sciences, University of Copenhagen, 2200 Copenhagen N, Denmark; ^5^ Department of Public Health, University of Copenhagen, 1014 Copenhagen K, Denmark; ^6^ Laboratory of Molecular Gerontology, National Institute on Aging, National Institutes of Health, Baltimore, MD 21224-6825, USA; ^7^ Research Centre for prevention and Health, Glostrup University Hospital, 2600 Glostrup, Denmark; ^8^ Department of Molecular Biology and Genetics, University of Aarhus, 8000 Aarhus C, Denmark

**Keywords:** vitality, mitochondrial respiration, reactive oxygen species, deoxyribonucleotides

## Abstract

Low vitality (a component of fatigue) in middle-aged and older adults is an important complaint often identified as a symptom of a disease state or side effect of a treatment. No studies to date have investigated the potential link between dysfunctional mitochondrial ATP production and low vitality. Therefore, we measured a number of cellular parameters related to mitochondrial activity in peripheral blood mononuclear cells (PBMCs) isolated from middle-aged men, and tested for association with vitality. These parameters estimate mitochondrial respiration, reactive oxygen species (ROS) production, and deoxyribonucleotide (dNTP) balance in PBMCs. The population was drawn from the Metropolit cohort of men born in 1953. Vitality level was estimated from the Medical Outcomes Study Short Form 36 (SF-36) vitality scale. We found that vitality score had no association with any of the mitochondrial respiration parameters. However, vitality score was inversely associated with cellular ROS production and cellular deoxythymidine triphosphate (dTTP) levels and positively associated with deoxycytidine triphosphate (dCTP) levels. We conclude that self-reported persistent low vitality is not associated with specific aspects of mitochondrial oxidative phosphorylation capacity in PBMCs, but may have other underlying cellular dysfunctions that contribute to dNTP imbalance and altered ROS production.

## INTRODUCTION

The Medical Outcomes Study Short Form 36 (SF-36) vitality scale used in this study is a frequently cited and validated subscale or component of the multi-component SF-36 fatigue scale. The SF-36 vitality scale focuses on energy level to capture differences in subjective well-being and disease burden [1]. SF-36 vitality is strongly associated with other components of the SF-36 fatigue scale, such as SF-36 physical functioning, mental health, bodily pain scores, and decreased sleep quality (all P < 0.001) [2, 3]. Of the eight SF-36 scales, the SF-36 vitality scale is the best at discriminating between levels of health, even in the healthiest subgroups [2]. In addition, SF-36 vitality shows significant association with all three components of another fatigue scale, the Fatigue Symptom Inventory (FSI) [4]. Therefore, SF-36 vitality is often used as a surrogate for fatigue. Fatigue/low vitality is a common complaint among older adults and one of the most frequent reasons for physician visits in general practice [1, 2, 4, 5]. Fatigue/low vitality is often a symptom of underlying psychiatric or medical illness and is increasingly seen as an early indicator of frailty, caused by increased vulnerability in multiple biologic and physiologic systems [5]. Previous studies find that the SF-36 vitality level is associated with several clinical conditions including anemia, congestive heart failure, chronic obstructive pulmonary disease, chronic fatigue syndrome, mortality risk, and with negative outcomes including inability to work, job loss, and hospitalization [1, 6]. It is likely that several cellular factors contribute to development of low vitality, and mitochondrial dysfunction is an obvious culprit due to its function in ATP production. Surprisingly, there have been no studies to assess a potential role of defects in mitochondrial oxidative phosphorylation, or other mitochondrial-related defects, in human vitality. Towards this end, we measured several mitochondrial-related cellular parameters in peripheral blood mononuclear cells (PBMCs) to assess potential associations of mitochondrial activities with vitality score. Such analysis may give insight into mechanisms underlying low vitality or lead to biomarkers for diseases that have low vitality as a core symptom. PBMCs are commonly used in peripheral biomarker discovery research since they are easily isolated from the blood. In addition, accumulating evidence suggests that alterations in markers in peripheral serum or cells are identifiable footprints that reflect dysfunctions occurring elsewhere, such as in the brain [7]. For example, changes in psychiatric disorders are not only found in the brain, but also in peripheral markers, such as cytokines [8, 9]. Moreover, PBMCs have been shown to be a suitable cell model for mitochondrial dysfunction and oxidative stress in biomarker discovery for Alzheimer's disease, fibromyalgia and chronic fatigue syndrome [10-12].

**Figure 1:**
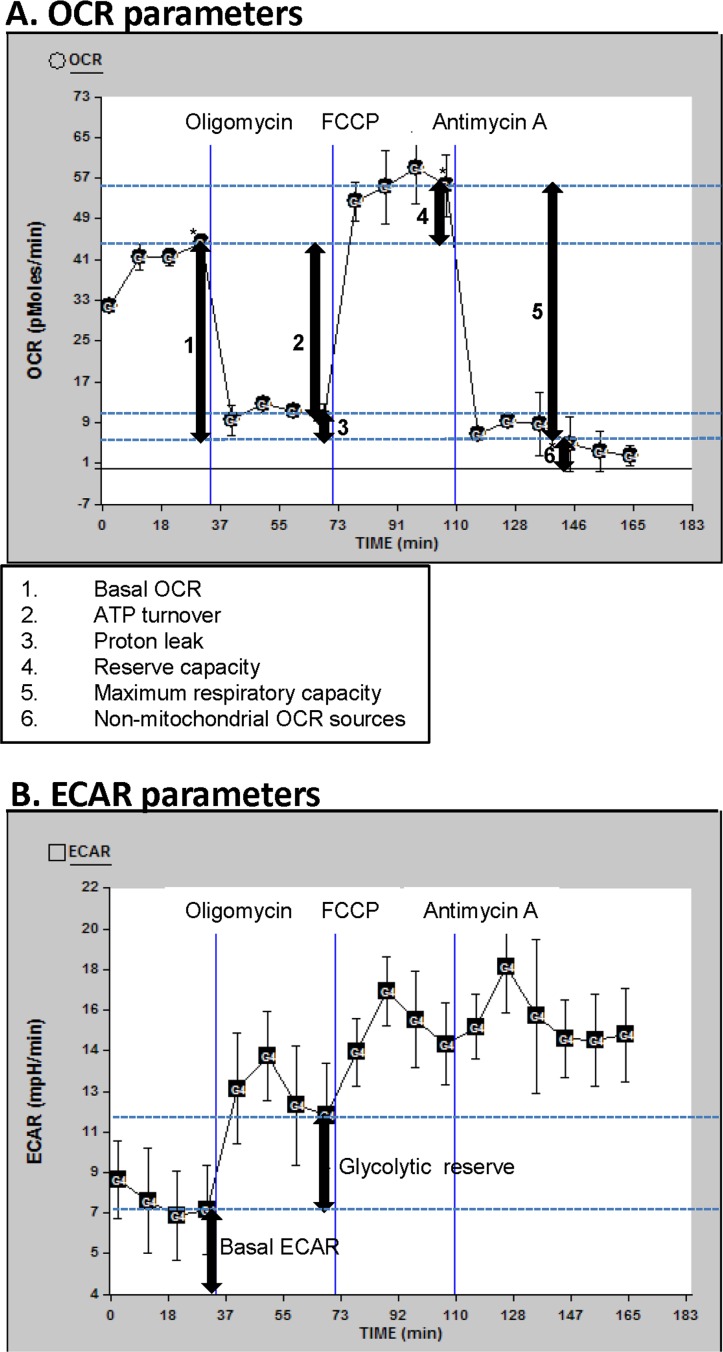
OCR and ECAR profiles Description of the oxygen consumption rate (OCR) (**A**) and extracellular acidification rate (ECAR) (**B**) parameters determined in this manuscript; for this illustration, data curves generated from a randomly selected participant in this study were used. Basal OCR and ECAR were measured (simultaneously), followed by measurements taken after sequential addition of oligomycin (1 μM), FCCP (0.3 μM) and antimycin A (2 μM). The fourth rate measurement after the beginning of the run and after each drug was added was used for the OCR and ECAR measurements, to allow for equilibration (rates 4, 8, 12, and 16 as shown). The first drug added was oligomycin. This drug inhibits ATP synthesis by blocking the proton channel of ATP synthase (Complex V). This results in a decrease in OCR to the extent to which the cells are using mitochondria to generate ATP. The remaining OCR is due to proton leak across the mitochondrial membrane and non-mitochondrial sources such as various desaturase and detoxification enzymes. There will be a concomitant increase in ECAR as the cells revert to glycolysis to meet their energy demands (this parameter is termed glycolytic reserve). The next drug added was FCCP, which is an uncoupling agent that disrupts ATP synthesis by transporting hydrogen ions across the mitochondrial membrane instead of the proton channel of ATP synthase. This leads to a rapid consumption of oxygen without the generation of ATP. ECAR may increase slightly beyond the existing ECAR as the cells continue to attempt to maintain their energy balance by using glycolysis to generate ATP. The final drug added was antimycin A, a complex III inhibitor. This causes the flow of electrons through the electron transport chain to cease. Therefore, consumption of oxygen is drastically reduced. Again, the ECAR may also increase slightly if necessary to maintain cellular energy balance.

Since mitochondria produce the majority of cellular energy (in the form of ATP), and mitochondrial dysfunction can result in metabolic and degenerative diseases [13], it is possible that mitochondrial dysfunction could contribute to fatigue, low vitality and the decline in functional status with aging. Cells generate ATP and biosynthetic precursors through a combination of oxidative and glycolytic metabolism. ATP production capacity can be measured conveniently by the Seahorse Bioscience's extracellular flux analyzer through detection of the oxygen consumption rate (OCR), to quantify mitochondrial respiration, and the extracellular acidification rate (ECAR), an indicator of glycolysis. Bioenergetics analysis suggests that measurement of mitochondrial respiratory fluxes give more information about the ability to make ATP than do measurements of membrane potential or ATP levels [14-16]. In this report, our interest lies with mitochondrial respiration capacity and plasticity under mitochondrial respiration stress or changing ATP demands, not with the cellular level of ATP at time of blood withdrawal. Therefore, our focus in this respect was to look for correlations of vitality score with mitochondrial respiration and glycolysis parameters, by using well-defined modulators of mitochondrial respiration (oligomycin, FCCP and antimycin A; see below and Figure 1).

We also measured cellular reactive oxygen species (ROS) production, in light of their relationship with mitochondrial function. Mitochondria are a significant source of ROS, however, there are also significant non-mitochondrial sources of endogenous ROS, including endoplasmic reticulum and peroxisomes [17]. Oxidative stress reflects the balance between the production rate of ROS and the removal rate of ROS by the antioxidant system. If oxidative stress is increased, it can damage DNA, RNA, proteins, and lipids and can lead to cell death and senescence and can have deleterious effects on all major organ systems [18, 19]. ROS are associated with the aging process and many age-associated diseases. ROS are also important signaling molecules, having crucial roles in normal physiological functioning. ROS-related diseases can stem from lack of ROS (such as certain autoimmune disorders) or surplus of ROS (such as cardiovascular and neurodegenerative diseases) [20].

In addition, we examined cellular deoxyribonucleotides (dNTPs) levels. dNTPs are the precursors used by polymerases for replication and repair of nuclear and mitochondrial DNA. The accuracy of these events requires sufficient amounts and correct balance of the four dNTPs. Imbalance of dNTP pools leads to genomic instability and is harmful to the cell and can lead to disease [21, 22]. In fact, recent studies have led to the hypothesis that there is an interconnected relationship between dNTP metabolism, genome stability, aging and cancer [23]. Several enzymes involved in biosynthesis of dNTPs are located in the mitochondria membrane. Studies suggest that mitochondrial dysfunctions affect these metabolic pathways and limit the available levels of cytosolic dNTPs [24]. Our rational for measuring dNTP ratios in this study is this connection to mitochondrial dysfunction, and that, unlike OCRs, ECARs and ROS, dNTPs can be measured on frozen samples and thus would serve well as biomarkers of fatiguing illnesses.

We performed association analysis between participant vitality scores and several OCR and ECAR parameters, as well as cellular ROS production and cellular dNTP levels. The objective of our study was to provide association-based evidence for or against underlying molecular defects that contribute to subjective feelings of low vitality, as well as search for potential biomarkers towards ailments that have low vitality as a core symptom.

## RESULTS

### Mitochondrial respiration and glycolysis are not associated with vitality

We measured mitochondrial and glycolytic respiratory parameters as indicators of mitochondrial respiration and glycolysis, as described in detail in the legend of Figure 1. Six OCR parameters (Figure 1A) and two ECAR parameters (Figure 1B) were determined and compared to vitality score. The two ECAR parameters that we report here (basal ECAR and glycolytic reserve) were chosen because they are the most informative with respect to the contribution of glycolysis to cellular energy production. We did not report changes in ECAR after FCCP or antimycin A addition since there is typically very little change in ECAR due to these treatments. None of the OCR or ECAR parameters had a significant association with vitality score (Table 1: Figures S1 and S2). This data indicates that in PBMCs the reported mitochondrial and glycolytic respiratory parameters are not linked to vitality. Note that non-mitochondrial OCR is generated by non-mitochondrial NADPH oxidases and other enzymes such as desaturase and detoxification enzymes. This rate has been subtracted out so that all other OCR readings represent mitochondrial OCRs (see Figure 1A). Because of inherent noise such as differences in cell size or number of mitochondria, absolute rates (basal OCR and basal ECAR) can be rather different from one individual to another and thus many participants are needed to help alleviate this problem. Other parameters such as reserve capacity are internally normalized and so are not as sensitive to differences in absolute rates. However, since there is no trend of basal OCR, or any of the other OCR parameters, with vitality (Table 1 and Figure S1) we feel that the N of 40 we have is sufficient, in that more participants would not change the null OCR-vitality association. In the case of ECARs, there appears to be an inverse trend of vitality with basal ECAR (Table 1 and Figure S2A) and no trend with glycolytic reserve (Table 1 and Figure S2B).

**Table 1:** Association of the cellular variables with vitality

Covariate	N	Mean ± SD	*P* value	Pearson r	Effect
**ROS production**	97	4.95 ± 1.75	0.036*	−0.213	−2.14
**dTTP**	79	2.28 ± 1.28	0.030*	−0.245	−3.60
**dATP**	79	4.76 ± 2.24	0.121	−0.176	−1.48
**dGTP**	79	4.01 ± 1.73	0.488	−0.079	−0.86
**dCTP**	34	2.35 ± 1.41	0.048*	0.342	4.20
**Basal OCR^†^**	40	26.84 ± 10.15	0.975	0.005	0.010
**ATP turnover^†^**	40	23.60 ± 10.03	0.86	0.029	0.058
**Reserve capacity^†^**	40	11.61 ± 9.65	0.827	0.036	0.075
**Maximum capacity^†^**	40	38.40 ± 16.60	0.888	0.023	0.028
**Proton leak^†^**	40	3.40 ± 2.50	0.647	−0.075	−0.609
**Non-mitochondrial OCR^†^**	40	21.57 ± 13.48	0.348	0.153	0.230
**Basal ECAR^††^**	40	5.18 ± 2.84	0.178	−0.217	−1.558
**Glycolytic reserve^††^**	40	3.96 ± 1.65	0.785	0.045	0.548

*P* values and Effect values were determined by regression analysis with vitality score as the dependent variable and the cellular parameter as the covariate. Pearson r values were generated using Pearson's correlation analysis. Since we are considering only a bivariate association, Pearson correlation analysis gives the same P value as regression analysis. “Effect” is defined as the effect of one unit increase in the variable on vitality (see Methods). This data analysis is also displayed graphically in Supplemental. SD = standard deviation. Units: ROS production, pmol/min/million cells; dNTPs, pmol/million cells; OCRs (labeled with symbol^†^), pmol oxygen/min; ECARs (labeled with symbol^††^), mpH/min.

† OCR and

†† ECAR parameters are described in Figure 1. As can be seen in Figure 1, the non-mitochondrial OCR has been subtracted out so that all other OCRs represent mitochondrial OCRs.

* Statistically significant (*P <* 0.05).

### Production of ROS and levels of dTTP and dCTP are associated with vitality

We also measured whole cell ROS levels and whole cell dNTP levels; these parameters are potential indicators of disturbances in signal transduction, genomic stability or mitochondrial function. We found that ROS production was inversely associated with vitality score (Table 1; Figure S3). We also found that dTTP level was inversely associated with vitality score, whereas dCTP level showed a positive association (Table 1; Figure S4), suggesting that dNTP balance is linked to vitality. To assess links among the parameters we measured in this study, we preformed association analysis (Table 2). From the above data, one might expect to see an association of ROS with dTTP (since both of these measures were inversely associated with vitality); we did not detect a significant association, however there appears to be a trend (P = 0.112, Pearson r = 0.225; Table 2). We did however find that the dATP level was significantly associated with ROS production and with dTTP, as well as with the other purine, dGTP (Table 2). Future experiments could pursue a potential mechanism that involves dNTP imbalance and cellular ROS production in development of low vitality or fatigue. ROS and dNTP levels are known to be affected by mitochondrial dysfunction, and thus one might also expect to see some association of these measures with the mitochondrial respiratory measures. We found that none of the mitochondrial OCR parameters correlated with ROS production or any of the dNTP levels. It should be noted that ROS and dNTP levels are measured in whole cell, as this is most convenient in terms of biomarker/high throughput measurements and cell material availability. It is possible that mitochondrial ROS and dNTP levels in the mitochondria (excluding cytoplasmic concentrations) would be associated with mitochondrial respiratory parameters; isolating mitochondria to test for this would not be feasible in our study due to limitation of material (cells isolated from blood).

**Table 2:** Associations among the cellular variables

Variable		dTTP	dATP	dGTP	dCTP	Basal OCR^†^	ATP turnover^†^	Reserve capacity^†^	Maximum capacity^†^	Proton leak^†^	Non-mitochondrial OCR^†^	Basal ECAR^††^	glycolytic reserve^††^
**ROS production**	*P* *r* N	0.112 0.225 51	0.029* 0.306 51	0.143 0.208 51	0.746 0.065 27	0.059 0.408 22	0.073 0.389 22	0.6 0.119 22	0.148 0.319 22	0.452 0.169 22	0.333 0.216 22	0.319 0.223 22	0.65 0.102 22
**dTTP**	*P* *r* N		< 0.0001* 0.454 79	0.139 0.168 79	ND	0.107 0.277 35	0.103 0.28 35	0.711 0.065 35	0.226 0.21 35	0.913 0.019 35	0.737 0.059 35	0.341 −0.166 35	0.356 0.161 35
**dATP**	*P* *r* N			< 0.0001* 0.566 79	ND	0.974 0.006 35	0.992 −0.002 35	0.302 0.179 35	0.522 0.112 35	0.617 0.088 35	0.717 −0.064 35	0.243 −0.203 35	0.939 0.013 35
**dGTP**	*P* *r* N				ND	0.716 −0.064 35	0.588 −0.095 35	0.463 0.128 35	0.823 0.039 35	0.351 0.163 35	0.539 −0.108 35	0.948 −0.011 35	0.709 0.065 35
**dCTP**	*P* *r* N					ND	ND	ND	ND	ND	ND	ND	ND
**Basal OCR^†^**	*P* *r* N						< 0.0001* 0.964 40	0.012* 0.394 40	< 0.0001* 0.844 40	0.271 0.178 40	0.089 0.272 40	0.217 0.2 40	0.0008* 0.511 40
**ATP turnover^†^**	*P* *r* N							0.002* 0.476 40	< 0.0001* 0.87 40	0.611 −0.083 40	0.011* 0.397 40	0.3 0.168 40	0.0001* 0.575 40
**Reserve capacity^†^**	*P* *r* N								< 0.0001* 0.826 40	0.079 −0.281 40	0.049* 0.314 40	0.574 0.092 40	0.002* 0.473 40
**Maximum capacity^†^**	*P* *r* N									0.728 −0.057 40	0.027* 0.35 40	0.284 0.174 40	< 0.0001* 0.587 40
**Proton leak^†^**	*P* *r* N										0.014* −0.387 40	0.498 0.11 40	0.182 −0.216 40
**Non-mitochondrial OCR^†^**	*P* *r* N											0.159 0.227 40	0.0007* 0.515 40
**Basal ECAR^††^**	*P* *r* N												0.020* 0.366 40

*P* values and Pearson r (*r*) values were generated using Pearson's correlation analysis. ND refers to “not determined” due to lack of overlapping participants for the corresponding two data sets. Units: ROS production, pmol/min/million cells; dNTPs, pmol/million cells; OCRs (labeled with symbol^†^), pmol oxygen/min; ECARs (labeled with symbol^††^), mpH/min.

† OCR and

†† ECAR parameters are described in Figure 1.

* Statistically significant (*P <* 0.05).

### Multiple associations among respiratory parameters-proof of principle

The association analysis of Table 2 also revealed that several of the respiratory parameters are strongly associated with each other. This is not surprising based on the principles of the flux analyzer pharmaceutical profiling approach [25] (see Methods section), and its use in cell culture-based experiments [15, 16, 25, 26]. However, we are the first to show that these relationships hold true in PBMCs in a participant cohort, giving proof of principle for the pharmaceutical profiling approach in participant PBMCs. Basal OCR and non-mitochondrial OCR were both positively associated with ATP turnover, reserve capacity, maximum capacity, and glycolytic reserve. These data imply that, in a population of middle aged men, higher basal mitochondrial and non-mitochondrial respiration are linked to higher ability of the cells to respond to altered energy demands. The above pharmaceutically-induced (non-basal) respiratory parameters (ATP turnover, reserve capacity, maximum capacity and glycolytic reserve) were also all positively associated with each other (Table 2). Proton leak was only associated with non-mitochondrial OCR (inversely). Basal ECAR was only associated with glycolytic reserve.

## DISCUSSION

Our study is the first to examine mitochondrial respiration in PBMCs with respect to vitality. Our bioenergetics analysis showed that none of the OCR parameters was associated with vitality score, indicating that the capacity of mitochondrial ATP production may not be related to vitality. In addition, there was no association of vitality score with basal ECAR or with the oligomycin-induced ECAR shift (glycolytic reserve), suggesting that alternative ATP generation by glycolysis (as needed when mitochondrial ATP generation is reduced) is also not linked to vitality. Although no studies before us have investigated potential links of mitochondrial dysfunction to vitality, several studies have reported mitochondrial dysfunctions in fatiguing illness [27-30], by examining levels of electron transport chain components and by estimating mitochondrial oxidative phosphorylation by way of ATP profiles. However, two recent studies in which the activities of the electron transport chain components were measured (in PBMCs in one of the studies) concluded that chronic fatigue syndrome patients have normal oxidative phosphorylation capacity [31, 32].

Our study is the first to report an association between ROS production in PBMCs and SF-36 vitality. This result is consistent with findings from illnesses that have low vitality and fatigue as core symptoms. Reports indicate that chronic fatigue syndrome and fibromyalgia are accompanied by higher oxidized low density lipoproteins and elevated protein carbonyl levels in the blood [10, 33-36]. In addition, elevated levels of 8-hydroxy-deoxyguanosine, a marker of oxidative damage to DNA, had been found in the urine of chronic fatigue patients [37]. These findings suggest that increased ROS levels may be involved in the development of these fatiguing disorders [38]. At moderate concentrations, ROS participate in the regulation of many cellular processes, including differentiation, proliferation, apoptosis, and cytoskeletal regulation [20, 39-44]. ROS initiates and regulates these pathways by directly interacting with critical signaling molecules, such as ATM, PI3 kinase, iron regulatory protein and redox factor 1 (for complete current review of this so called “oxidative interface” pathway activation process see Ray et al. [45]). Many of the ROS-mediated responses actually protect the cell against oxidative stress and re-establish redox homeostasis and regulate mitochondrial energy production [43]. We speculate that our reported association of ROS with vitality score could involve signaling pathway imbalance. Along these lines, studies in humans have shown that oxidative changes in plasma thiol/disulphide redox status are correlated with aging-related pathophysiological processes, such as immune response, thyroid function, and cognitive function [20, 40, 46]. However, many correlation studies, including our current study, cannot imply cause and effect.

The dTTP level was inversely associated with vitality score, yet the dCTP level showed a significant association with vitality score in the opposite direction. Therefore, our data suggests that low vitality may be linked to both a lower dCTP and higher dTTP level. Thus, going forward, a potentially useful predictor of low vitality/fatigue or related illnesses could be low dCTP/dTTP ratio. However this would have to be verified in future studies. We did not specifically measure this ratio in this study because, due to lack of sufficient material, dCTP measurements were done on a different set of participants (N of 34; see Table 1) than for the other three dNTPs (N of 79; see Table 1); we had to set aside 34 frozen samples that had not been measured yet, specifically for dCTP-only measurements to allow us to obtain data from all four dNTPs. One could also look for alterations in the level or activity of deoxycytidylate (dCMP) deaminase. This enzyme catalyzes the conversion of dCMP to dUTP in the *de novo* synthesis of thymidine nucleotides and, via this activity, is believed to play a key role in regulating the dCTP/dTTP balance in the cell [22]. The physiological consequences of dCMP deaminase defects or imbalance in dCTP/dTTP are not well understood. However there is evidence for a role of dCMP deaminase in genomic integrity and tumorigenicity [23]. Speculation based on the above associations would be that dCMP deaminase is overactive in low vitality (high fatigue) patients (since this enzyme catalyzes a step in dTTP synthesis and the dTTP levels we measured in this study were inversely associated with vitality). Other important enzymes important in dNTP balance that could be examined, with respect to vitality, are dihydroorotate dehydrogenase (DHODH) and ribonucleotide reductase (RNR) [22, 24]. DHODH is important in *de novo* synthesis of pyrimidines and is located in the inner mitochondrial membrane and its activity is influenced by the activity of the electron transport chain. RNR catalyzes the formation of deoxyribonucleotides from ribonucleotides and can be inhibited by nitric oxide that is created by mitochondrial nitric oxide synthase (mtNOS) located in the inner mitochondrial membrane. The activity of mtNOS is linked to the mitochondrial membrane potential. We note that our measurement of total cellular dNTP levels and total ROS levels are not designed to differentiate the source of these molecules (mitochondrial versus cytosolic); this would require more elaborate work (involving isolation of mitochondria), not amenable to a cohort study. However, typically mitochondrial dNTP pools represent at most 10% of the total cellular pools [21]. This suggests that the dNTP imbalance that we observe, as a function of vitality, could stem from a defect in cytosolic, as opposed to mitochondrial, dNTP pathways. From the above data, we cannot say whether the ROS and dNTP associations with vitality have any mitochondrial-defect link.

We found no association of the mitochondrial OCR parameters with ROS production or dNTP levels (Table 3). Data from previous studies suggest that mitochondrial dysfunction may be linked to oxidant levels [47-50] and dNTP imbalance [24, 51]. However, those studies did not measure mitochondrial oxygen flux respiratory parameters, but rather focused on other measures of mitochondrial dysfunction, that are linked to respiration, such as oxidized protein carbonyls [52], peroxidation of membrane phospholipids [47-49], and mitochondrial DNA mutations [51]. An exception is a recent report by Furda et al. [53] in which treatment of mouse embryonic fibroblasts with H_2_O_2_ resulted in a reduction in the OCR parameters ATP turnover and reserve capacity. However, sustained H_2_O_2_ treatment was necessary to see this effect. To get a more complete picture of bioenergetics, with respect to vitality, further activities could be examined, such as electron delivery to the respiratory chain, substrate transport into the cell, and ATP export to the cytoplasm. We did not measure ATP concentration as our focus was on mitochondrial ATP generation dysfunction as measured via oxygen flux respiratory parameters. However, Myhill et al. [29] recently reported a strong correlation between an “ATP profile” test measure (which includes ATP concentration) in neutrophils and the severity of chronic fatigue syndrome. However, ATP-related experiments would also have to assess whether observed changes in cellular ATP reflects dysfunctional mitochondria or an independent change in the metabolism of adenine nucleotides [15].

It is known that several physiological/bioenergetic conditions regulate the OCR levels, and the OCR parameters are interrelated: for example, basal OCR level is controlled to a large degree by ATP turnover (as evident by our strong correlation between these two parameters; Table 2), and partly by proton leak (both of which consume the proton-motive force generated by the substrate oxidation) [15]. Therefore, differences in ATP demand between individuals (for example, due to differences in protein synthesis or plasma membrane ion cycling [15, 26, 54]) strongly influence basal OCR levels. OCRs can also be altered by difference in levels of pyruvate or glucose; this was controlled by careful preparation of incubation medium containing defined levels of pyruvate and glucose. The maximum OCR reports the maximum activity of electron transport that is achievable by the cells under the assay conditions (artificial decoupling of electron transport from ATP synthesis by FCCP). A decrease in maximum OCR is a strong indicator of mitochondrial dysfunction in level or efficiency of electron transport (hence, our strong correlation of maximum capacity with basal OCR, ATP turnover and reserve capacity). Reserve capacity (also called spare-respiratory capacity) is an important diagnostic of the ability of the cell to response to changes in ATP demand. As is obvious in Figure 1A, it represents the maximum OCR above the basal OCR and so indicates how close to its bioenergetic limit the cell is operating. It was correlated with basal OCR, ATP turnover, and maximum capacity, which indicated that all these parameters are linked to the cells ability to respond to changes in ATP demand. The glycolysis parameter, basal ECAR (Figure 1B), did not correlate with any mitochondrial respiratory parameters; however it did correlate with the other measured glycolysis (ECAR) parameter, glycolytic reserve. The glycolytic reserve correlated with all the OCR parameters except for proton leak. Previous studies have validated, in cultured cells, the concept of switching to glycolysis for generation of ATP when the mitochondrial ATP generation is dysfunctional [16, 55]; our OCR-glycolytic reserve correlations validates this concept in PBMCs and thus reiterate that in a population a person with high mitochondrial consumption of oxygen (in their PBMCs) would also tend to have a high ability to switch to glycolytic generation of ATP when the mitochondrial ATP generation is dysfunctional. A thorough discussion of the links between the mitochondrial and glycolytic bioenergetic measures can be found in other articles [15, 16, 56]. Interestingly the non-mitochondrial OCR showed significant associations with several mitochondrial OCR parameters. Future in-vitro experiments will be needed to extent this observation, by examining specific non-mitochondrial oxygen consuming reactions in PBMC or other cells types that may alter with induced changes in respiratory parameters. These include cyclooxygenases, lipoxygenases and potentially NADPH oxidases and nitric oxide synthases, all of which generate either reactive lipids or oxidants such as hydrogen peroxide, which can directly modify mitochondrial function. In should be noted that the term non-mitochondrial OCR may not be completely accurate, as there may be a component of this OCR parameter that stems from mitochondrial sources other than oxidative phosphorylation; therefore “non-oxidative phosphorylation OCR” may be a more accurate term, however, “non-mitochondrial” is almost exclusively used in the literature in this context [15, 16, 57], and therefore we use this term. Moreover, in immune cells, such as in PBMCs, the predominant antimycin A-insensitive OCR is due to oxygen consuming immune cell activities such as NADPH oxidases [15]. Due to the above relationships of the respiratory parameters one would expect that if one respiratory parameter was associated with vitality, then other respiratory parameters would also be associated with vitality. We in fact saw that none of the OCR or ECAR parameters was associated with vitality score. We conclude that mitochondria respiratory dysfunction at the level of the above respiratory parameters are not factors in human vitality.

Our data suggests that low vitality is not linked to defects in mitochondrial respiration or glycolysis, but may have other underlying molecular defects that impact ROS levels and dNTP balance. We speculate that high ROS production and dNTP pool imbalance may interact with development of low vitality. These measures could be pursued as components of early biomarker strategies for fatiguing illness. The ROS association data supports the practice of antioxidant intake as a method to lift or maintain energy levels in healthy individuals and suggests that antioxidant intake could be examined as part of an intervention strategy in patients that need to maintain high vitality for quick recovery.

## METHODS

### Cohort selection and vitality score evaluation

The study population was drawn from the Metropolit Cohort of men born in 1953 [58], as part of the Copenhagen Aging and Midlife Biobank (CAMB) data collection [59]. The CAMB data collection took place at The National Research Centre for the Working Environment in 2010 and included blood tests, clinical examinations (height, weight, waist measurements, blood pressure, and physical performance tests), cognitive tests, and a postal questionnaire on health, health behavior, depressive mood, and social factors. Vitality was measured by the Medical Outcomes Study Short Form 36 (SF-36) vitality scale, which consists of 4 items scored from 1-6. The scores from the 4 items were summed together and transformed [100 × [mean ((7-score of item 1) + (7-score of item 2) + score of item 3 + score of item 4)−1]/5] to a scale ranging from 0-100. The above measures were performed on the CAMB cohort of 2487 persons as part of the CAMB study. For practical reasons the various cellular tests were performed on various population sizes, ranging from 34 to 97, depending on the test (see Table 1). None of the participants reported to have experienced myocardial infarction or angina pectoris in the past year. Unpublished data from our ongoing analysis has indicated that blood pressure (diastolic and systolic) and smoking have no significant association with vitality (N of 2427 and 205 respectively). This study was conducted according to the ethical principles of the Helsinki II declaration. All participant components of the project were approved by the Ethical Review Committee of the Capital Region of Copenhagen (H-A-2008-126). The specific project was approved by the Danish Data Protection Agency (No: 2011-41-6175). The project is not associated with any risk or harm to the participants.

### PBMC isolation and storage

PBMCs were isolated using BD Vacutainer Cell Preparation Tubes (CPT) containing sodium citrate (BD biosciences), according to the manufacturer's protocol. PBMC isolation was performed on 8 ml of blood sample per participant. Cells were counted by a cell counter (CASY^®^ cell counter, Roch Innovatis AG) and then diluted to 2 million cells per ml in PBS and aliquoted for the various tests (see below). The Casey counter revealed two main peaks representing platelets and the PBMCs. The PBMC peak was selected for counting. Isolated PBMCs were diluted to 2 million cells per ml in PBS and used immediately or aliquoted for the various tests (see below), as follows: fresh cells were used for bioenergetic (Seahorse XP analyzer) and ROS measurements. For the dNTP assay, 0.5×10^6^ million PBMCs were centrifuged in freezer tubes and the cell pellet was resuspended in 60% methanol and directly frozen and stored in liquid nitrogen. Several aliquots of cells, as available, were also frozen away for future use: the purified PBMCs were centrifuged and then the cell pellet resuspended in freezing medium (50% fetal bovine serum, 40% DMEM, 10% DMSO) in freezing tubes at 0.3×10^6^ cells per tube, and then frozen first in −80°C in a pre-cooled (4°C) freezing container overnight (-1°C/min cooling rate) and then moved for long term storage to liquid nitrogen.

ROS production. Whole cell hydrogen peroxide (H2O2) release (pmol H2O2 per minute per 106 cells) was measured in isolated PBMCs with Amplex Red (Molecular Probes) as a trapper of H2O2, catalyzed by horseradish peroxidase. The H2O2 reacts with Amplex Red in a 1:1 stoichiometry yielding the fluorescent compound resorufin (excitation 560 nm, emission 590 nm), which is stable once formed. Fluorescence was measured continuously with a spectrofluorometer equipped with temperature control (37°C) and stirring (SAFAS Xenius, Monaco). Isolated PBMCs were added to PBS buffer along with Amplex Red (0.05 mM) and horseradish peroxidase (12 U/ml). Superoxide dismutase (SOD) (90 U/ml) was added to convert the produced superoxide to H2O2. Subsequently succinate (5 mM) was added as substrate for mitochondrial ROS production, and digitonin (50 μg/ml) was added to permeabilize the cell membrane. This protocol was devised to establish the total cellular ROS production. Six different concentrations of H2O2 were included to establish a standard curve. All measures were corrected for background by subtraction of a no-PBMC control.

### dNTP levels

Whole cell levels of deoxyadenosine triphosphate (dATP), deoxycytidine triphosphate (dCTP), deoxyganosine triphosphate (dGTP) and deoxythymidine triphosphate (dTTP) were determined using the DNA polymerase assay previously described [51]. Cellular dNTPs were extracted from 0.5×10^6^ PBMCs with 60% methanol. Radioactivity was measured in a Tri-Carb 2900TR liquid scintillation counter (Packard) and normalized to pmol/1×10^6^ cells using a standard curve of known dNTP concentrations. Due to low values for dCTP, the test was performed again for only dCTP measurement, on the frozen material from the remaining participants. Thus, the participant set for dCTP was not the same as the participant set that was used for all the other three dNTPs.

### Bioenergetic parameters

An XF24 Analyzer (Seahorse Biosciences) was used to measure bioenergetic function, specifically oxygen consumption rates (OCRs; respiratory parameters that estimate the efficiency of mitochondrial respiration [16]) and extracellular acidification rates (ECARs; parameters that estimate of the level of glycolysis [16]), in PMBCs in 24 well dishes (XF24 V7, Seahorse Bioscience), in response to added pharmaceutical modulators of mitochondrial oxidative phosphorylation (oligomycin, FCCP and antimycin A; Figure 1). This method is based on a published pharmaceutical profiling approach [25]. The chemical concentrations and PBMC seeding density were determined by titration: final concentrations of 1 μM, 0.3 μM and 2 μM, for oligomycin, FCCP and antimycin A, respectively, in wells containing 300,000 cells, resulted in an optimal stress response (OCR shifts) profile (see Figure 1 for representative OCR and ECAR profiles).

### Statistical analysis

The relationship between PBMC parameters and vitality scores (Table 1) was estimated by regression analysis with vitality score as the dependent variable and the cellular parameter as the covariate; with a linear relationship between the two variables. The P value (Table 1) is the same whether we use regression or correlation analysis since we are considering simple bivariate associations. The effects of these parameters, defined as the change in vitality score when the cellular parameter is increased by one unit, were quantified using the regression coefficient and the p-value. This “effect” statistic must be considered in context of the scale and mean value for the covariant; a one unit increase will be more dramatic if the covariant mean value is smaller. The reporting of effect sizes facilitates the interpretation of the substantive, as opposed to the statistical, significance of a research result. Regression analysis was done using SAS version 9.2. Pearson r (Table 1), as well as the association analysis among the cellular parameters (Table 2) and the supplemental data figures (graphical representation of Table 1 data), were performed using Pearson's correlation analysis with GraphPad Prism 5 software (La Jolla, California, USA). P < 0.05 was considered significant.

## SUPPLEMENTAL DATA



## Abbreviations

PBMCs: peripheral blood mononuclear cells
dNTP: deoxyribonucleotide
SF-36: Medical Outcomes Study Short Form 36
CAMB: Copenhagen Aging and Midlife Biobank
OCR: oxygen consumption rate
ECAR: extracellular acidification rate
ROS: reactive oxygen species
