# Supplementary material for: Relationships between human vitality and mitochondrial respiratory parameters, reactive oxygen species production and dNTP levels in peripheral blood mononuclear cells
Source: Aging (Albany NY). 2013 Nov 30;5(11):850–64. doi: 10.18632/aging.100618 (PMC3868727; doi:10.18632/aging.100618)
Supplement: Supplementary file 1 [file aging-05-850-s001.pdf]

SUPPLEMENTAL DATA

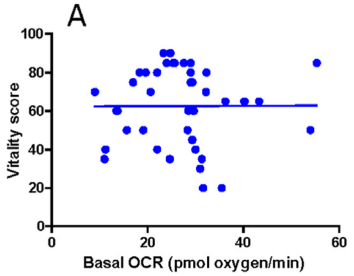

Number of XY Pairs	40
Pearson r	0.005204
95% confidence interval	-0.3069 to 0.3163
P value (two-tailed)	0.9746
P value summary	ns
Is the correlation significant? (alpha=0.05)	No
R square	2.708e-005

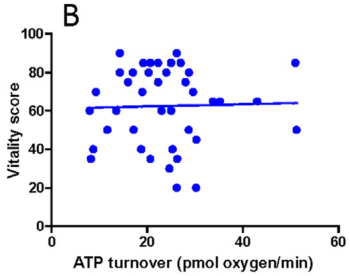

Number of XY Pairs	40
Pearson r	0.02871
95% confidence interval	-0.2854 to 0.3373
P value (two-tailed)	0.8604
P value summary	ns
Is the correlation significant? (alpha=0.05)	No
R square	0.0008245

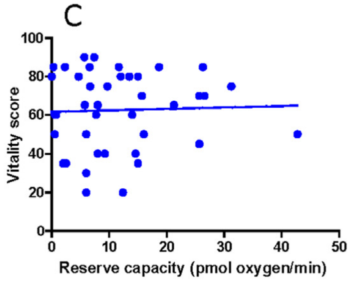

Number of XY Pairs	40
Pearson r	0.03564
95% confidence interval	-0.2790 to 0.3434
P value (two-tailed)	0.8272
P value summary	ns
Is the correlation significant? (alpha=0.05)	No
R square	0.001270

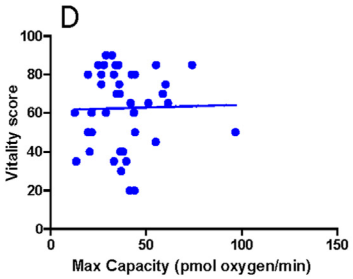

Number of XY Pairs	40
Pearson r	0.02304
95% confidence interval	-0.2906 to 0.3322
P value (two-tailed)	0.8878
P value summary	ns
Is the correlation significant? (alpha=0.05)	No
R square	0.0005307

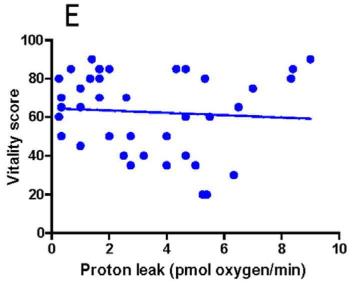

Number of XY Pairs	40
Pearson r	-0.07474
95% confidence interval	-0.3775 to 0.2425
P value (two-tailed)	0.6467
P value summary	ns
Is the correlation significant? (alpha=0.05)	No
R square	0.005586

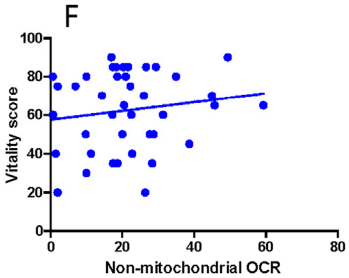

Number of XY Pairs	40
Pearson r	0.1525
95% confidence interval	-0.1670 to 0.4430
P value (two-tailed)	0.3475
P value summary	ns
Is the correlation significant? (alpha=0.05)	No
R square	0.02325

Figure S1. Correlation analysis between vitality score and OCR parameters. Graphical representation of the analyzed OCR parameter data of Table 1. Correlation analysis was done between vitality scores and (A) basal OCR, (B) ATP turnover, (C) reserve capacity, (D) maximum capacity, (E) proton leak, (F) non-mitochondrial OCR. Vitality score scale ranges from 0-100. Pearson's correlation analysis was performed using GraphPad Prism 5.0 software, with a cutoff of $P < 0.05$ considered as significant.

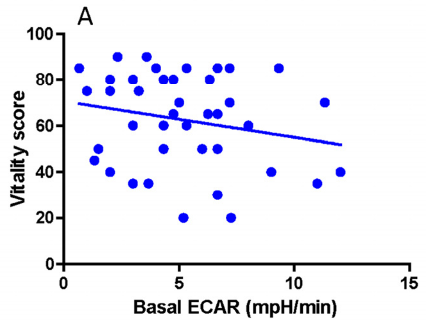

Number of XY Pairs	40
Pearson r	-0.2173
95% confidence interval	-0.4953 to 0.1012
P value (two-tailed)	0.1781
P value summary	ns
Is the correlation significant? (alpha=0.05)	No
R square	0.04720

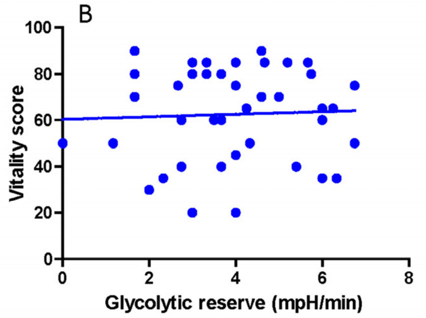

Number of XY Pairs	40
Pearson r	0.04460
95% confidence interval	-0.2707 to 0.3513
P value (two-tailed)	0.7846
P value summary	ns
Is the correlation significant? (alpha=0.05)	No
R square	0.001989

Figure S2. Correlation analysis between vitality score and ECAR parameters. Graphical representation of the analyzed ECAR parameter data of Table 1. Correlation analysis was done between vitality scores and (**A**) basal ECAR, (**B**) glycolytic reserve. Vitality score scale ranges from 0-100. Pearson's correlation analysis was performed using GraphPad Prism 5.0 software, with a cutoff of $P < 0.05$ considered as significant.

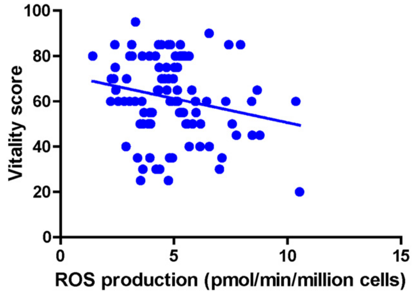

Number of XY Pairs	97
Pearson r	-0.2133
95% confidence interval	-0.3959 to -0.01442
P value (two-tailed)	0.0359
P value summary	*
Is the correlation significant? (alpha=0.05)	Yes
R square	0.04550

Figure S3. Correlation analysis between vitality score and ROS production. Graphical representation of the analyzed reactive oxygen species (ROS) data of Table 1. Correlation analysis was done between vitality scores and whole cell hydrogen peroxide (H_2O_2) release (pmol H_2O_2 per minute per 10^6 cells). Vitality score scale ranges from 0-100. Pearson's correlation analysis was performed using GraphPad Prism 5.0 software, with a cutoff of $P < 0.05$ considered as significant.

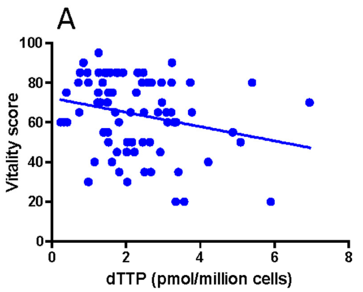

Number of XY Pairs	79
Pearson r	-0.2446
95% confidence interval	-0.4419 to -0.02481
P value (two-tailed)	0.0298
P value summary	*
Is the correlation significant? (alpha=0.05)	Yes
R square	0.05984

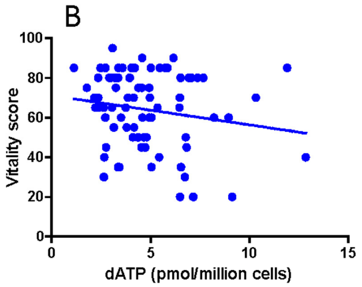

Number of XY Pairs	79
Pearson r	-0.1759
95% confidence interval	-0.3822 to 0.04706
P value (two-tailed)	0.1209
P value summary	ns
Is the correlation significant? (alpha=0.05)	No
R square	0.03095

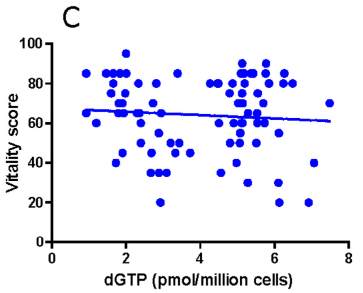

Number of XY Pairs	79
Pearson r	-0.07910
95% confidence interval	-0.2951 to 0.1446
P value (two-tailed)	0.4884
P value summary	ns
Is the correlation significant? (alpha=0.05)	No
R square	0.006256

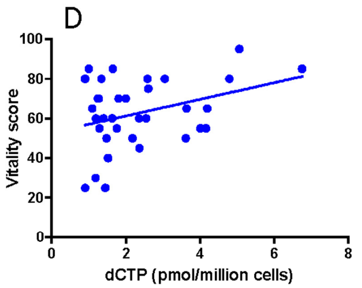

Number of XY Pairs	34
Pearson r	0.3420
95% confidence interval	0.004246 to 0.6097
P value (two-tailed)	0.0477
P value summary	*
Is the correlation significant? (alpha=0.05)	Yes
R square	0.1170

Figure S4. Correlation analysis between vitality score and dNTP levels. Graphical representation of the analyzed dNTPs data of Table 1. Specifically, correlation analysis was done between vitality scores and (**A**) deoxythymidine triphosphate (dTTP), (**B**) deoxyadenosine triphosphate (dATP), (**C**) deoxyganosine triphosphate (dGTP) and (**D**) deoxycytidine triphosphate (dCTP). Vitality score scale ranges from 0-100. Pearson's correlation analysis was performed using GraphPad Prism 5.0 software, with a cutoff of $P < 0.05$ considered as significant.
